# The Mediating Role of General Self-Efficacy in Health Self-Management and Psychological Stress Among Older Adults in Shanghai: A Structural Equation Modeling Analysis

**DOI:** 10.3390/healthcare13010046

**Published:** 2024-12-30

**Authors:** Rong Du, Ruilong Wu, Jing Li, Jiayan Jiang, Hengyi Zhou, Jingrong Gao, Qi Zhao

**Affiliations:** 1 School of Public Health, NHC Key Laboratory of Health Technology Assessment, Fudan University, Shanghai 200032, China; dur22@m.fudan.edu.cn (R.D.); 19211020066@fudan.edu.cn (J.J.); zhouhengyi@scdc.sh.cn (H.Z.); 2 Shanghai Institute of Occupational Disease for Chemical Industry, Shanghai 200041, China; 3 Zhongshan Street Community Health Service Center, Shanghai 201613, China; 4 Shanghai Tenth People’s Hospital, Shanghai 200040, China; 5 Shanghai Municipal Center for Disease Control and Prevention, Shanghai 200336, China; 6 Shanghai Municipal Center for Health Promotion, Shanghai 200040, China

**Keywords:** older adults, psychological distress, general self-efficacy, health self-management, structural equation modeling

## Abstract

Background: Given the demographic shift towards an older population, it is crucial to understand the determinants and implications of psychological distress among this demographic group. This study explores the relationship between health self-management and psychological distress in older Chinese adults, focusing on the mediating role of general self-efficacy (GSE). Methods: We conducted a cross-sectional study in five districts of Shanghai from 1 November to 31 December 2021, employing a random cluster sampling method. Data were collected using the Health Self-Management Scale, the General Self-Efficacy Scale (GSES), and the Kessler-10 Scale. Out of 2050 participants approached, 1905 completed the survey, resulting in a response rate of 91.76%. Data analysis included correlation studies, hierarchical linear regression, and structural equation modeling. Results: The average Kessler-10 score among elderly individuals in Shanghai was 15.59 ± 6.35. Of these, 22.10% had mild psychological distress, 11.92% had moderate stress, and 5.30% had severe stress. We found significant negative correlations between psychological distress and health self-management (r = −0.34, *p* < 0.05) and GSE (r = −0.26, *p* < 0.05). Health self-management had a total effect of −0.284 (95%CI: −0.330 to −0.234) on psychological distress, with GSE mediating approximately 20.42% of this effect. Conclusions: GSE plays a significant mediating role between health self-management and psychological distress. These findings highlight the importance of promoting health self-management and GSE to alleviate psychological distress among older adults in Shanghai. This approach could enhance holistic health outcomes and improve the quality of life within this growing population segment.

## 1. Introduction

Mental disorders have emerged as a significant contributor to the global disease burden [1]. Between 1990 and 2019, the estimated number of cases of mental disorders increased from 654.8 million to 970.1 million, representing a 48.1% rise [2]. Concurrently, as global aging accelerates, a growing number of elderly individuals are experiencing periods of heightened risk for mental disorders. This vulnerability is often compounded by a high prevalence of chronic diseases and diminishing physical functioning, leading to psychological distress marked by generalized symptoms such as stress, anxiety, and depression [3,4]. China, the world’s most populous nation, has seen its proportion of elderly residents rise from 4.6% in 2011 to 13.5% in 2021. In particular, Shanghai’s elderly population reached 5.42 million in 2021, accounting for 36.3% of the city’s total population [5]. In light of these demographic shifts, it is imperative to examine the current state of psychological distress among China’s elderly and to identify effective interventions to mitigate their suffering.

The traditional medical model is a paternalistic framework characterized by the authoritative physician being afforded maximum discretion by the patient [6]. However, with the aging population, the rising prevalence of chronic diseases, and the increasing pressure on healthcare resources, health self-management has emerged as one of the effective measures to reduce healthcare burdens and meet individual health needs. Individuals are also gaining more autonomy in managing their health. Health self-management, based on social cognitive theory, self-efficacy theory, and self-determination theory, emphasizes leveraging social and familial resources and the individual initiative to monitor and regulate one’s health [7,8]. The Health Self-Management Scale, developed by Zhao and Huang in 2011 [9], evaluates the capabilities of health self-management across three dimensions: behavior, cognition, and environment. Health self-management behavior refers to individuals’ actions, such as adopting specific dietary practices, engaging in physical exercise, and employing disease-coping strategies, to prevent illness and protect their health. Health self-management cognition pertains to individuals’ health-related beliefs and their sense of self-efficacy. The health self-management environment encompasses the utilization of public health resources, the creation of a supportive health environment, and the seeking and leveraging of available support [9]. Health self-management has been extensively applied in managing chronic diseases such as asthma, diabetes, arthritis, and hypertension [10,11]. However, its application in addressing psychological distress remains insufficiently examined in the existing literature. Research indicates that engaging in effective self-management practices—including dietary modifications, regular physical activity, and fostering strong interpersonal relationships—can mitigate negative emotions and stress, thereby improving individual well-being and quality of life, and also contributing to enhanced mental health and social welfare [12,13,14]. Among workers, the implementation of health self-management strategies has been shown to reduce symptoms of anxiety and depression [15]. Furthermore, in the elderly population, those exhibiting milder depressive symptoms appear more inclined to participate in health self-management activities [16].

Albert Bandura initially proposed the concept of domain-specific self-efficacy in 1977 [17]. Building upon his framework, researchers later expanded the scope to include broad self-efficacy, which refers to an individual’s confidence in their capability to handle diverse demands or novel situations [18,19]. General self-efficacy (GSE) specifically focuses on cultivating a stable and extensive sense of personal competency, which is essential for managing various stressful scenarios [20]. Environmental factors, individual perceptions, and behaviors, including health self-management practices, shape this overarching confidence. Both the environment and individual perceptions significantly influence GSE [17,21]. From a behavioral perspective, experiences, whether successful or not, impact an individual’s GSE. Emotionally, low levels of GSE are linked with depression, anxiety, helplessness, and reduced self-esteem [20]. Highlighting its value in mental health contexts, GSE is recognized as a robust predictor of psychological well-being and has demonstrated potential in alleviating psychological distress [22].

In summary, existing research has established a correlation between health self-management and GSE, and their association with psychological distress. Yet, the potential mediating role of GSE between these factors remains unclear. Consequently, it is essential to measure these relationships within the population of older adults in China and to implement effective interventions to alleviate psychological distress in this group, and ultimately enhance their mental health. This study aims to elucidate the interactions among health self-management, GSE, and psychological distress through structural equation modeling. This study proposes the following hypotheses: (1) Health self-management is inversely associated with psychological distress; (2) GSE serves as a partial mediator in the relationship between health self-management and psychological distress.

## 2. Materials and Methods

### 2.1. Study Design and Participants

The study was conducted in Shanghai from 1 November 1 to 31 December 2021, utilizing a cluster random sampling methodology. Five districts were selected for inclusion in the study—two urban and three suburban—based on their economic status and demographic profiles. From each district, one community was randomly chosen as the research site. The participants were residents aged 60 and older who visited health service centers in these communities. Data were collected through structured questionnaires. The inclusion criteria for participants were as follows: (1) age 60 years or older, (2) mental clarity sufficient to understand and complete the questionnaire, (3) ability to communicate effectively, and (4) provision of informed consent. The exclusion criteria included: (1) patients suffering from severe medical conditions such as paralysis, Alzheimer’s disease, or deafness, (2) individuals experiencing communication impairments, and (3) those unwilling to participate in the study. With reference to prior research [23], which identified that 29.2% of individuals had K10 scores equal to or greater than 16, the sample size was calculated taking into account a margin of error of 10% of the proportion (p), a confidence level (α) of 0.05, and an anticipated non-response rate of 10%. Consequently, the final sample size required for the study was determined to be 1025 participants.

### 2.2. Measurement and Model

Face-to-face interviews were conducted using questionnaires administered by 20 uniformly trained community physicians, under the direct supervision and quality control of the researcher. The questionnaire included general sociodemographic data and three validated scales.

#### 2.2.1. Individual Information

Demographic data collected included age, gender (men, women), residential area (urban or suburban), marital status (married, single/divorced/widowed), level of education, monthly personal income, living situation (alone or with others), life satisfaction (higher or lower), personal behavior habits, and presence of chronic diseases (yes or no). Educational attainment was categorized as “high school and above”, “middle school”, or “elementary school and below”. Monthly personal income was segmented into “< 3000 Chinese yuan (CNY)”, “3000–4999 CNY”, and “≥ 5000 CNY”. Personal behavior included variables such as smoking, alcohol consumption, physical activity, and others.

#### 2.2.2. Psychological Distress

The Kessler Psychological Distress Scale (K10) [24] was employed to evaluate the emotional states of the participants over the last 4 weeks. The scale comprises 10 items, each featuring five response options: never, rarely/seldom, sometimes, most of the time, and always. The detailed items are provided in the supplementary materials Table S1. Scores on the K10 range from a minimum of 10 to a maximum of 50, with higher scores indicating severe psychological distress among respondents. The scoring categories are as follows: 10 to 15 suggests the individual is likely well, 16 to 21 indicates mild psychological distress, 22 to 29 suggests moderate psychological distress and 30 to 50 indicates severe psychological distress [25]. This scale has been demonstrated to possess strong reliability and validity within Chinese populations [26]. For the present sample, Cronbach’s α was calculated at 0.951.

#### 2.2.3. Health Self-Management

The Health Self-Management Scale, initially developed by Zhao and Huang in 2011 [9], served as the instrument to evaluate participants’ health self-management capabilities. This scale comprises three subscales: health self-management behavior (14 items), health self-management environment (10 items), and health self-management cognition (14 items), resulting in a total of 38 items. The detailed items are provided in the supplementary materials Table S2. Responses were recorded using a 5-point Likert scale, where each item ranged from 1 to 5. The aggregate of these three dimensions constitutes the overall health self-management score, with higher scores indicating better health self-management among adults. For the present study, the internal consistency of the scale, as assessed by Cronbach’s α, was 0.961.

#### 2.2.4. General Self-Efficacy

The General Self-Efficacy Scale (GSES), developed by Schwarzer and Jerusalem in 1997 [20], was utilized to assess the GSE of older adults. This instrument comprises 10 items, each rated on a four-point Likert scale that spans from 1 (“completely nonconforming”) to 4 (“completely conforming”). The detailed items are provided in the supplementary materials Table S3. The composite score, obtained by summing the scores of all items, ranges from 10 to 40, where higher scores denote increased self-efficacy. In the current study, the Cronbach’s α was 0.944, indicating high reliability.

### 2.3. Statistical Analysis

Statistical analysis was conducted using SPSS 25.0 and AMOS 23.0. To address missing data, multiple imputation techniques were employed, enhancing the robustness of the results across imputed data sets. The K10 scores were compared across different subgroups using t-tests and ANOVA, while Spearman’s correlation coefficient was utilized to investigate associations among health self-management, GSE, and psychological distress. Hierarchical linear regression analyses were performed to assess the incremental R-squared values contributed by the added variables, with K10 scores as the dependent variable. The models incorporated variables as follows: Model 1 included demographic characteristics, Model 2 incorporated health self-management, and Model 3 added GSE. Statistically significant variables from the linear regression analyses were then included in the structural equation model (SEM) to explore the structural relationships among multiple latent variables and to assess the direct, indirect, and total effects of the independent variables on the dependent variable. The relationships among latent variables were examined using SEM and analyzed through robust maximum likelihood estimation (MLR) [27]. Health self-management was evaluated across environmental, behavioral, and cognitive dimensions. GSE and psychological distress were each measured using a respective 10-item scale. The goodness-of-fit indices for the SEM (χ2/df < 5, GFI, AGGI, CFI, TLI > 0.90, RMSEA < 0.08) confirmed that the model met the requisite criteria. Further, the mediating role of GSE between health self-management and psychological distress was explored using a bias-corrected nonparametric percentile bootstrap method with a sample size of 5000. Bias-corrected 95% confidence intervals for all effects were calculated, and statistical significance was assessed using two-tailed probability values < 0.05.

## 3. Results

### 3.1. Sociodemographic Characteristics

A total of 2076 questionnaires were distributed, with 1905 respondents (91.76%) completing them. The participants’ average age was 70.99 ± 5.56 years, with women comprising 58.32% of the sample. Geographically, 51.18% of the participants resided in urban areas, while only 8.40% lived alone. Regarding educational attainment, 33.81% had received at least a high school education. Additionally, 35.28% of the elderly’s monthly income was over 5000 CNY. Satisfaction with life was high, with 63.62% of participants reporting high levels of satisfaction. Chronic diseases were reported by 53.54% of the sample. Behavioral patterns indicated that 75.80% of the elderly had never smoked and 79.79% had never consumed alcohol, as shown in Table 1.

### 3.2. Psychological Distress, GSE, and Health Self-Management

The average scores for the K10, GSES, and Health Self-Management Scale were 15.59 (SD = 6.35), 28.58 (SD = 5.05), and 150.75 (SD = 23.99), respectively, as shown in Table 2. Based on the predefined cutoff criteria for the K10, 60.68% of the participants were deemed to likely have good mental health. Conversely, 22.10%, 11.92%, and 5.03% of older adults exhibited mild, moderate, and severe psychological distress, respectively.

The mean K10 score for women (15.96 ± 6.38) was significantly higher than that for men (15.08 ± 6.28, t = −3.00, *p* < 0.01). Older individuals tended to have higher K10 scores (t = −0.31, *p* < 0.01). Married older adults had a mean K10 score of 15.25 (SD = 6.20), which was significantly lower than those who were single, divorced, or widowed, who scored 17.27 (SD = 6.79). Higher K10 scores were observed among older adults who lived alone (t = 4.30, *p* < 0.01) and those who communicated infrequently with their children (t = −5.65, *p* < 0.01). Additionally, individuals with higher education (F = 12.47, *p* < 0.01) and higher monthly personal income (F = 13.29, *p* < 0.01) exhibited relatively lower K10 scores. Lifestyle factors also influenced K10 scores: older adults who smoked (t = −2.98, *p* < 0.01), or consumed alcohol (t = −3.09, *p* < 0.01) presented lower scores compared to their counterparts. Conversely, individuals with chronic diseases displayed higher K10 scores (t = 2.23, *p* = 0.03).

### 3.3. Correlations Among Variables

**Figure 1:**
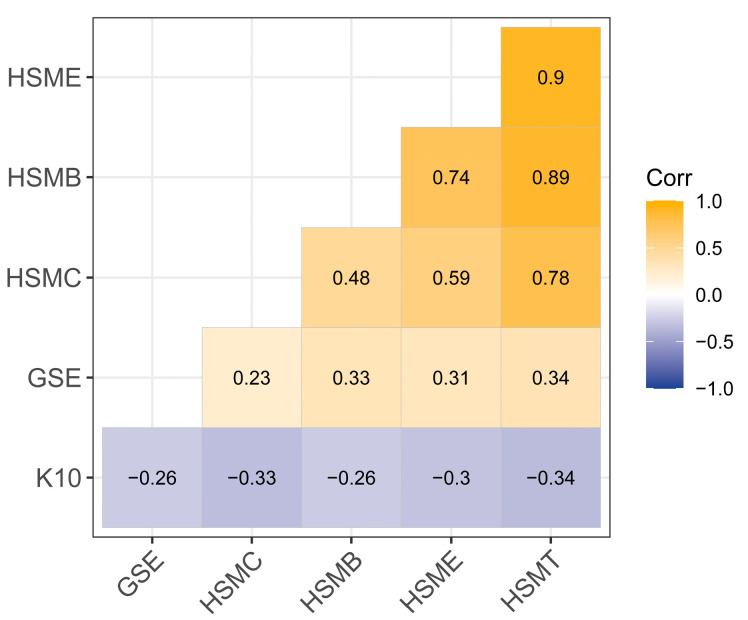
The correlation among K10 score, general self-efficacy, and health self-management (*p* < 0.01). Abbreviations: K10, K10 score; GSE, General self-efficacy; HSMC, Health self-management cognition; HSME, Health self-management environment; HSMB, Health self-management behavior; HSMT, Health self-management total scores.

Figure 1 illustrates a significant correlation between health self-management, GSE, and K10 scores in elderly individuals. Specifically, the K10 score exhibited a significant negative correlation with both GSE and health self-management. This suggests that severe psychological distress in older adults is associated with lower levels of health self-management and GSE.

### 3.4. Analysis of Variables Using Regression

Table 3 presents a hierarchical linear regression analysis. The dependent variable in this analysis is the K10 score. Model 1 investigates the impact of demographic characteristics on the K10 score among the elderly, accounting for 11.8% of the variance. This model includes independent variables such as gender, age, place of residence, marital status, education, income, living arrangements, behavioral habits, and others. Model 2 examines the relationship between health self-management and the K10 score. Model 3 subsequently includes GSE, which explains 20.9% of the variance in the K10 score. Results indicate a significant negative correlation between health self-management and the K10 score, with health self-management contributing 7.5% to the variance. Furthermore, GSE also demonstrates a significant negative correlation with the K10 score, influencing the impact of health self-management.

### 3.5. Analysis of Mediating Effects

**Figure 2:**
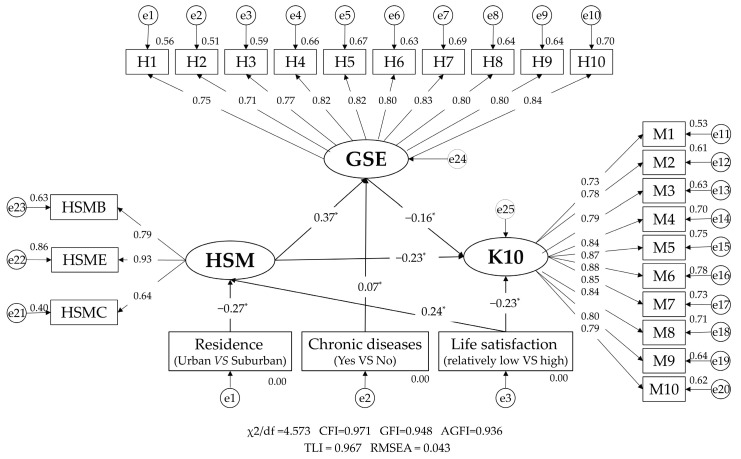
The standardized structural equation model of health self-management on K10 score with general self-efficacy as a mediator. Abbreviations: K10: K10 score; GSE: General self-efficacy; HSMC: Health self-management cognition; HSME: Health self-management environment; HSMB: Health self-management behavior; HSM: Health self-management; H: General self-efficacy item; M: K10 score item. * *p* < 0.01.

The standardized SEM validated the direct, indirect, and total effects linking health self-management, GSE, and K10 scores. The fit indices (χ^2^/df = 4.573, CFI = 0.971, GFI = 0.948, AGFI = 0.936, TLI = 0.967, RMSEA = 0.043) demonstrated a satisfactory model fit. The factor loadings for the three dimensions of health self-management—behavior, environment, and cognition —were 0.79, 0.93, and 0.64, respectively, all statistically significant. Similar significance was observed in the factor loadings for each dimension of the K10 scores and GSE. The total effect of health self-management on K10 scores was −0.284 (95% CI: −0.330 to −0.234), with a direct effect of −0.226 (95% CI: −0.275 to −0.171). Additionally, the indirect effect of health self-management on K10 scores, mediated through general self-efficacy (GSE), was −0.058 (95% CI: −0.079 to −0.039), which accounted for 20.42% of the total effect, as shown in Figure 2 and Table 4. Interestingly, health self-management also served as a mediator in the relationship between life satisfaction and K10 scores, mediating approximately 22.64% of this effect.

## 4. Discussion

This research explored the dynamics of health self-management, GSE, and psychological distress. The results demonstrated a significant negative correlation between health self-management and psychological distress. Furthermore, GSE was identified as a partial mediator in the relationship between health self-management and psychological distress.

This study found that the K10 score for elderly individuals in Shanghai was 15.59 ± 6.35, with prevalence rates of mild, moderate, and severe psychological distress at 22.10%, 11.92%, and 5.30%, respectively, consistent with findings from Australia [3]. However, participants aged 70 and above exhibited higher K10 scores, likely due to the greater proportion of women (57.80%) in this age group, as women generally report higher K10 scores than men [28]. Residing in urban areas, having high levels of life satisfaction, and being free from chronic diseases were identified as protective factors against psychological distress in older adults. These protective effects could be attributed to higher economic status, more abundant medical resources, and relatively lower stress levels experienced by urban-dwelling older adults [29,30]. Life satisfaction may influence psychological distress indirectly through the mediation of health self-management. One possible explanation is that individuals with lower life satisfaction may lack the motivation to maintain or adhere to self-management plans, thereby increasing psychological distress [31]. Furthermore, older adults with chronic diseases, particularly those with multiple symptoms or comorbid conditions, exhibited significantly higher levels of psychological distress [3,32].

The results from structural equation modeling indicated that higher levels of health self-management among older adults were associated with reduced psychological distress, corroborating previous research [12]. GSE was identified as playing a mediating role in this relationship. Physiologically, effective health self-management behaviors, such as regular physical exercise, can activate or regulate various physiological mechanisms. This leads to the production of neurotransmitters and neurotrophic factors, which in turn help to alleviate psychological distress [33]. Additionally, individuals with higher life satisfaction tend to have greater motivation to seek and utilize social and family support, thereby enhancing health self-management and overall well-being [34,35]. The mediating influence of GSE is further evident across the environmental, cognitive, and behavioral dimensions of health self-management. Studies have shown that environments supportive of health self-management—characterized by guidance from community health centers, family support, regular health monitoring, and peer education—significantly enhance an individual’s GSE [36,37,38]. High levels of GSE are associated with a positive and optimistic mindset, which is inversely related to psychological distress [20]. The cognitive aspect of health self-management involves acquiring the knowledge and skills necessary to manage negative emotions that arise during the process, which in turn enhances individual confidence [39]. However, the cognition of health self-management and general self-efficacy are both related and distinct. The former emphasizes beliefs and efficacy specifically related to health, while the latter focuses on confidence when facing challenges and difficulties. In terms of behavior, the GSE of older adults is shaped by their experiences within the realm of health self-management. Positive experiences tend to enhance GSE, while repeated failures may significantly diminish it, consequently heightening psychological distress [40]. This dynamic underscores the complexity of how GSE impacts the psychological well-being of older adults engaged in health self-management.

Overall, health self-management offers a promising approach to alleviate psychological distress among older adults, representing a shift from the traditional medical model toward one that emphasizes personal autonomy. This study is the first to investigate the mediating role of GSE between health self-management and psychological distress in this demographic, providing valuable insights for reducing psychological distress among older adults. First, the increasing aging population makes psychological distress in older adults a significant concern. Second, enhancing health self-management and GSE through multidimensional approaches is crucial for reducing psychological distress in older adults. Collaborative efforts involving society, communities, and families are vital in creating a supportive environment for effective self-management. Initiatives in health education and promotion should aim to improve understanding of health self-management and GSE among the elderly. Furthermore, establishing realistic and achievable milestones in health self-management processes, coupled with timely evaluation and reinforcement of self-management behaviors, enhances individuals’ successful experiences, thereby boosting GSE. The ultimate goal is to consistently reduce psychological distress among the elderly, enabling them to maintain good mental health and enjoy a high quality of life. Finally, it is important to emphasize that health self-management applies to all older adults, both with and without chronic conditions. However, for those with chronic diseases, a greater focus should be placed on disease self-management, such as adhering to prescribed medication regimens, monitoring symptoms, maintaining a balanced diet, and engaging in appropriate physical activity. For those without chronic conditions, the emphasis should be on health promotion and disease prevention.

This study has several limitations. Firstly, the structural equation model constructed in this study represents only one of the potential pathways among the variables, and the cross-sectional design constrains the ability to draw causal conclusions between them. Additionally, this study, conducted in Shanghai, included only older adults who visited community health centers, potentially resulting in a healthier sample. Future studies should include more diverse regions and less accessible populations to improve generalizability.

## 5. Conclusions

This study has clarified the complex interplay between health self-management and psychological distress, emphasizing the mediating role of GSE. This research enhances the theoretical framework regarding the impact of health self-management on psychological distress, providing important implications for the creation of targeted and effective interventions designed to alleviate psychological distress among older adults. It is essential to initiate multisectoral and multifaceted strategies that focus on improving environmental, cognitive, and behavioral factors related to health self-management and GSE, thereby reducing psychological distress in this demographic.

## Figures and Tables

**Table 1:** Distribution of K10 scores across subgroups of elderly adults in Shanghai (*n* = 1905).

Variables	*n*	%	M	SD	t/F	*p* Value
Gender					−3.00	<0.01
Men	794	41.68	15.08	6.28		
Women	1111	58.32	15.96	6.38		
Age (years)						
60–69	931	48.87	15.14	5.91	−0.31	<0.01
≥70	974	51.13	16.03	6.71		
Residence					−1.62	0.11
urban	975	51.18	15.82	6.23		
suburban	930	48.82	15.35	6.47		
Marital Status					−4.95	<0.01
In marriage	1580	82.94	15.25	6.20		
single/divorced/widowed	325	17.06	17.27	6.79		
Education level					12.47	<0.01
Elementary school and below	597	31.34	16.66	7.31		
Middle school	664	34.86	15.15	5.94		
High school and above	644	33.81	15.06	5.65		
Personal monthly income (CNY)					13.29	<0.01
<3000	364	19.11	16.57	7.27		
3000~4999	869	45.62	15.92	6.48		
≥5000	672	35.28	14.64	5.46		
Life satisfaction					12.67	<0.01
relatively low	693	36.38	18.09	7.06		
high	1212	63.62	14.17	5.41		
Living alone					4.30	<0.01
Yes	160	8.40	17.65	6.77		
No	1745	91.60	15.41	6.28		
Communication with children					−5.65	<0.01
Frequently	1082	56.80	14.87	5.85		
Infrequently	823	43.20	16.55	6.84		
Smoking					−2.98	<0.01
Yes	461	24.20	14.83	6.03		
No	1444	75.80	15.84	6.43		
Drinking					−3.09	<0.01
Yes	385	20.21	14.73	6.05		
No	1520	79.79	15.81	6.41		
Chronic diseases					2.23	0.03
Yes	1020	53.54	15.90	6.38		
No	885	46.46	15.25	6.29		

Abbreviations: M, mean; SD, standard deviation.

**Table 2:** K10 score, general self-efficacy, and health self-management scores of older adults in Shanghai.

Variables		M	SD
K10 scores		15.59	6.35
General Self-efficacy		28.58	5.05
Health Self-Management ability	Behavior	50.28	11.41
	Environment	38.85	8.05
	Cognition	61.63	8.45
	Total scores	150.75	23.99

Abbreviations: M, mean; SD, standard deviation.

**Table 3:** Hierarchical linear regression analysis of the K10 score of older adults in Shanghai.

		Model 1	Model 2	Model 3
Block1 Sociodemographic characteristics				
(Intercept)		17.509	29.842	32.968
Gender (Men vs. Women)		−0.296	−0.133	−0.168
Age (years) (60–69 vs. ≥ 70)		0.258	0.017	−0.051
Residence (Urban vs. Suburban)		−0.612 ^a^	−1.362 ^b^	−1.401 ^b^
Marital Status (In marriage vs. single/divorced/widowed)		1.220 ^a^	0.992 ^a^	0.846
Education level (Elementary school and below vs. Middle School)		−0.820 ^a^	−0.658	−0.433
Elementary school and above vs. High school and below		−0.565	−0.126	0.169
Personal monthly income (CNY) (< 3000 vs. 3000–4999)		−0.052	−0.072	0.034
< 3000 vs. ≥ 5000		−0.511	−0.649	−0.492
Life satisfaction (relatively low vs. high)		−3.420 ^b^	−2.720 ^b^	−2.645 ^b^
Living alone (Yes vs. No)		−0.200	−0.163	−0.038
Communication with children (Frequently vs. Infrequently)		1.053 ^b^	0.251	0.223
Smoking (Yes vs. No)		0.741	0.788	0.786
Drinking (Yes vs. No)		0.708	0.440	0.402
Chronic diseases (Yes vs. No)		−0.701 ^a^	−0.719 ^b^	−0.613 ^a^
Block2 Health Self-Management Ability			−0.080 ^b^	−0.069 ^b^
Block3 General Self-Efficacy				−0.177 ^b^
R^2^		0.118	0.193	0.209
ΔR^2^		0.118	0.075	0.016

Note: ^a^ *p* < 0.05; ^b^ *p* < 0.01; R^2^, coefficient of determination.

**Table 4:** The standardized total, indirect, and direct effects of health self-management and life satisfaction on K10 score with general self-efficacy as a mediator (*n* = 1905).

Model Pathways		Point Estimate	SE	95%CI
Total effect	HSM → K10	−0.284	0.024	−0.330~−0.234
	Life satisfaction → K10	−0.296	0.023	−0.341~−0.253
Indirect effect	HSM → GSE → K10	−0.058	0.010	−0.079~−0.039
	Life satisfaction → HSM → K10	−0.067	0.009	−0.086~−0.051
Direct effect	HSM → GSE	0.372	0.024	0.323~0.417
	HSM → K10	−0.226	0.027	−0.275~−0.171
	GSE → K10	−0.156	0.025	−0.205~−0.107
	Life satisfaction → HSM	0.236	0.023	0.192~0.281
	Life satisfaction → K10	−0.229	0.023	−0.275~−0.186
Proportion of the effect of HSM on K10 score				
Mediated by general self-efficacy				20.42%
Direct effect				79.58%
Proportion of the effect of life satisfaction on K10 score				
Mediated by health self-management				22.64%
Direct effect				77.36%

Abbreviations: K10, K10 score; GSE, General self-efficacy; HSM, Health self-management.

## Data Availability

The data presented in this study are available on request from the corresponding author. The data are not publicly available due to privacy.

## Supplementary Materials

The following supporting information can be downloaded at: https://www.mdpi.com/article/10.3390/healthcare13010046/s1, Table S1: K10 Score Assessment of Older Adults in Shanghai; Table S2: Health Self-Management Ability Assessment of Older Adults in Shanghai; Table S3: General Self-Efficacy Assessment of Older Adults in Shanghai.
