# Supplementary material for: The Mediating Role of General Self-Efficacy in Health Self-Management and Psychological Stress Among Older Adults in Shanghai: A Structural Equation Modeling Analysis
Source: Healthcare (Basel). 2024 Dec 30;13(1):46. doi: 10.3390/healthcare13010046 (PMC11720252; doi:10.3390/healthcare13010046)
Supplement: Supplementary file 1 [file healthcare-13-00046-s001.zip › healthcare-3341654-supplementary.pdf]

Supplementary appendix

This supplementary appendix includes detailed items for the K10 scores, health self-management, and general self-efficacy evaluation.

Table S1. K10 Score Assessment of Older Adults in Shanghai.

Table S2: Health Self-Management Ability Assessment of Older Adults in Shanghai.

Table S3. General Self-Efficacy Assessment of Older Adults in Shanghai.

These questions concern how you have been feeling over the past 30 days. Please reflect on your feelings and experiences over the past 30 days and place a "√" in the appropriate box for each item:

Table S1. K10 Score Assessment of Older Adults in Shanghai.

**Questions**	**None of the time**	**A little of the time**	**Some of the time**	**Most of the time**	**All of the time**
1. During the last 30 days, about how often did you feel tired out for no good reason?					
2. During the last 30 days, about how often did you feel nervous?					
3. During the last 30 days, about how often did you feel so nervous that nothing could calm you down?					
4. During the last 30 days, about how often did you feel hopeless?					
5. During the last 30 days, about how often did you feel restless or fidgety?					
6. During the last 30 days, about how often did you feel so restless you could not sit still?					
7. During the last 30 days, about how often did you feel depressed?					
8. During the last 30 days, about how often did you feel that everything was an effort?					
9. During the last 30 days, about how often did you feel so sad that nothing could cheer you up?					
10. During the last 30 days, about how often did you feel worthless?					

Please judge how much you agree or disagree with each statement and place a "√" in the corresponding box:

Table S2: Health Self-Management Ability Assessment of Older Adults in Shanghai.

**Behavior**	**None of the time**	**A little of the time**	**Some of the time**	**Most of the time**	**All of the time**
1. Regular three meals a day.					
2. Light diet.					
3. Scientifically allocate the proportion of breakfast, lunch, and dinner (eat well in the morning, eat enough in the middle of the day, and eat less in the evening).					
4. Pay attention to the nutritional balance of different kinds of food every day (more than 15 kinds, including onion/ginger/garlic/meat/vegetables).					
5. Drink at least 1200ml of water (about 6 cups) every day.					
6. Choose your exercise program, intensity, and duration according to your own or professional advice.					
7. Fully utilize exercise and rest venues and equipment.					
8. Exercise at least 3 times a week for at least 30 minutes each time.					
9. Exercise mainly with aerobic exercise (such as jogging, walking, tai chi, swimming, etc.)					
10. Timely and appropriately replenish fluids or electrolytes after exercise.					
11. Pay attention to labels, expiration dates, and usage instructions when taking medication.					
12. Seek medical attention in time when feeling unwell.					
13. Actively cooperate with medical staff when seeking treatment or care.					
14. Be able to clearly explain your condition to the doctor when seeking treatment or care.					
**Environment**	**None of the time**	**A little of the time**	**Some of the time**	**Most of the time**	**All of the time**
15. Clean and disinfect living utensils or equipment regularly.					
16. Keep the working or living environment neat and clean.					
17. Pay attention to food hygiene and safety.					
18. Create a good sleep environment (including light, sound, temperature, humidity, etc.)					
19. Improve indoor air quality in various ways when it is poor (such as opening windows for ventilation)					
20. Understand or utilize the appropriate medical insurance (e.g., extent of reimbursement, medicines, etc.)					
21. Pay attention to or make use of healthcare-related policies (e.g., new healthcare reform, new rural cooperative policy).					

22. Make appropriate use of the health care services around you (e.g., choose the appropriate organization for medical treatment)					
23. Take the initiative to seek help and support from others when needed.					
24. Fully utilize the acquired health knowledge.					
**Cognition**	**None of the time**	**A little of the time**	**Some of the time**	**Most of the time**	**All of the time**
25. Having good interpersonal relationships can promote health.					
26. Having a good living or working environment can promote health.					
27. Family members or friends can play a supervisory role in personal health management.					
28. Family members or friends can set a good example for one’s health management.					
29. Vaccination is an economical and effective measure to prevent certain infectious diseases.					
30. Reasonable use of health care medicines or equipment can improve one’s physical condition and alleviate one’s illness to a certain extent.					
31. Following healthcare professionals’ advice is one of the ways to maintain health.					
32. Diet, exercise, and mental attitude are important factors affecting health.					
33. Reducing smoking, increasing physical activity, and maintaining a balanced diet can reduce the incidence of cardiovascular and cerebrovascular diseases.					
34. Adhering to the treatment plan or advice prescribed by healthcare professionals.					
35. Being able to control oneself and not be influenced by others’ bad habits.					
36. Changing unhealthy lifestyles and adopting self-care behaviors.					
37. Be able to appropriately respond to health problems when they arise.					
38. Be able to identify harmful health behaviors, activities, or substances.					

Please judge how much you agree or disagree with each statement and place a "√" in the corresponding box:

Table S3. General Self-Efficacy Assessment of Older Adults in Shanghai.

Statements	Not at all true	Barely true	Moderately true	Exactly true
1. I can always manage to solve difficult problems if I try hard enough.				
2. If someone opposes me, I can find means and ways to get what I want.				
3. It is easy for me to stick to my aims and accomplish my goals.				
4. I am confident that I can deal efficiently with unexpected events.				
5. Thanks to my resourcefulness, I know how to handle unforeseen situations.				
6. I can solve most problems if I invest the necessary effort.				
7. I can remain calm when facing difficulties because I can rely on my coping abilities.				
8. When I am confronted with a problem, I can usually find several solutions.				
9. If I am in a bind, I can usually think of something to do.				
10. No matter what comes my way, I'm usually able to handle it.				
